# A Novel Mutation of *DAX-1* Associated with Secretory Azoospermia

**DOI:** 10.1371/journal.pone.0133997

**Published:** 2015-07-24

**Authors:** Lisha Mou, Nie Xie, Lihua Yang, Yuchen Liu, Ruiying Diao, Zhiming Cai, Honggang Li, Yaoting Gui

**Affiliations:** 1 Shenzhen Key Laboratory of Genitourinary Tumor, Shenzhen Domesticated Organ Medical Engineering Research and Development Center, Shenzhen Second People's Hospital, First Affiliated Hospital of Shenzhen University, Shenzhen, 518035, China; 2 Guangdong and Shenzhen Key Laboratory of Male Reproductive Medicine and Genetics, Institute of Urology, Peking University Shenzhen Hospital, Biomedical Research Institute, Shenzhen PKU-HKUST Medical Center, Shenzhen, 518036, China; 3 The Center of Reproductive Medicine, Tongji Medical College, Huazhong University of Science and Technology, Wuhan, 430030, China; Nanjing Medical University, CHINA

## Abstract

Secretory azoospermia is a severe form of male infertility caused by unknown factors. DAX-1 is predominantly expressed in mammalian reproductive tissues and plays an important role in spermatogenesis because *Dax-1* knockout male mice show spermatogenesis defects. To examine whether DAX-1 is involved in the pathogenesis of secretory azoospermia in humans, we sequenced all of the exons of *DAX-1* in 776 patients diagnosed with secretory azoospermia and 709 proven fertile men. A number of coding mutations unique to the patient group, including two synonymous mutations and six missense mutations, were identified. Of the missense mutations, our functional assay demonstrated that the V385L mutation caused the reduced functioning of DAX-1. This novel mutation (p. V385L) of DAX-1 is the first to be identified in association with secretory azoospermia, thereby highlighting the important role of DAX-1 in spermatogenesis.

## Introduction

The incidence of infertility is approximately 15% to 20% for couples of childbearing age, and about half of cases of infertility are caused by male factors [1, 2]. Secretory azoospermia, characterized by the absence of spermatozoa in semen, is a severe form of male infertility that affects approximately 1% of adult men in the general population [1, 2]. Previous studies have shown that genetic factors play an important role in IA [3].

DAX-1 (dosage-sensitive sex reversal, adrenal hypoplasia critical region on the X chromosome, gene 1; also called NROB1) is predominantly expressed in both male and female reproductive tissues [4–6]. Mutations in *DAX-1* cause X-linked adrenal hypoplasia congenita (AHC), a disorder characterized by primary adrenal failure, hypogonadotropic hypogonadism and azoospermia [7]. Accumulating evidence from studies of *Dax-1* knockout mice and those of AHC patients indicate that mutations in *DAX-1* may directly cause abnormalities in spermatogenesis [8–12]. In particular, previous studies have shown that gonadotropin is unsuccessful for the treatment of azoospermia patients with classic X-linked AHC, suggesting a direct effect of DAX-1 on spermatogenesis [13–16].

DAX-1 is a member of the orphan nuclear receptor family of transcription factors [17]. Previous *in vitro* and *in vivo* studies have shown that it represses the activity of androgen receptor (AR) by directly interacting with it [18–20]. As a result, certain sequence changes in DAX-1 may affect its interaction with AR and eliminate its repression of AR activity, ultimately resulting in abnormal spermatogenesis.

In the present study, we sequenced the exons of *DAX-*1 in 776 secretory azoospermia patients and 709 fertile men to estimate the association of this gene with secretory azoospermia. Two synonymous mutations and six missense mutations were identified that were unique to the patient group. Among them, the DAX-1 V385L mutation was present in one patient, resulting in the significantly reduced inhibition of the transcriptional activity of AR. Thus, we propose that this mutation in DAX-1 eliminates its repression of AR and that it potentially contributed to the onset of secretory azoospermia in this patient.

## Materials and Methods

### Patient samples

A total of 1,880 azoospermic patients were recruited for this study from the Center of Reproductive Medicine, Tongji Medical College, Huazhong University of Science and Technology from Jan 2007 to Oct 2011 [21]. Among of them, 776 Han Chinese patients fulfilled the following criteria for secretory azoospermia diagnosis: (1) no sperm detected in the pellets of semen samples on three different occasions; (2) no obstruction, inflammation or injury of the reproductive system or pelvic cavity; and (3) no karyotypic abnormality or Y chromosome microdeletion. A total of 709 fertile Han Chinese men from the Center of Physical Examination, Peking University Shenzhen Hospital were recruited as controls who had fathered at least one child without assisted reproductive techniques, such as IVF, ICSI or IMSI. After a panel re-sequencing study and quality control steps, 776 patients aged 24–46 years (average of 30.6 years) and 709 fertile men aged 29–51 years (average of 35.6 years) were available for further analysis.

### Ethics statement

Informed written consent was obtained from each subject, and the study was approved by the ethics committee of Peking University Shenzhen Hospital. All clinical investigations were conducted according to the principles of the Declaration of Helsinki.

### Panel re-sequencing study

Five micrograms of genomic DNA isolated from peripheral blood samples were sent to Beijing Genomics Institute at Shenzhen for exonic capture and sequencing. The capture procedure was performed in solution with a NimbleGen custom array (Roche NimbleGen, Madison, WI, USA) that is capable of enriching the exonic sequences of 654 infertility- or subfertility-related genes [21]. Most of these genes have been reviewed by Matzuk and Lamb [3]. Moreover, we selected other genes that have been shown to cause male reproductive defects in mouse models in studies published between November 2008 and December 2010. Panel re-sequencing was performed with an Illumina platform with 90 bp pair-end reads.

Fastq sequence files were aligned against the human reference genome (NCBI build 37.1, hg19) with SOAPaligner software (2.21). Duplicated paired-end reads were removed from the merged data sets. Single nucleotide variants that were not present in different from the hg19 reference genome were filtered out if they met any of the following criteria: a Phred-like quality score of ≤ 20, overall depth of ≤ 8×, estimated copy number of ≥ 2 or genomic distance between two adjacent variants of < 5 bp. In addition, the quality scores of both the major and minor alleles at heterozygous loci were at least 20. The variants were then annotated using an in-house functional prediction tool and were compared with dbSNP132 and 1000 Genomes databases (as of August 2010).

### Validation of novel missense mutations by Sanger sequencing

To validate the novel missense mutations identified by deep sequencing, PCR amplifications were carried out, and the PCR products were sequenced in both directions with a 3730 DNA analyzer (Applied Biosystems). The primers for PCR and Sanger sequencing validation of the *DAX-1* gene are listed in S1 Table.

### Western blot analysis and immunoprecipitation

For Western blot analysis, cells were washed once in PBS, resuspended in cell lysis buffer (38733, Sigma, Shanghai, China) with a protease inhibitor mixture containing PMSF and Cocktail, and then incubated on ice for 30 min. The lysates were then cleared by centrifugation at 12,000 rpm for 5 min, and the total protein was boiled for 5 min with SDS sample buffer.

Equal amounts of each protein sample were then separated on 10% SDS-polyacrylamide gels and transferred to nitrocellulose membranes. The membranes were blocked for 1 h at room temperature in 5% skim milk with 0.5% Tween-TBS (TBST) and then probed with an anti-AR (1:1000; sc-13062X, Santa Cruz, CA, USA) or anti-HA (1:1000; H3663; Sigma, Shanghai, China) antibody overnight at 4°C in TBST containing 5% skim milk. After being washed 3 times for 5 min with TBST, the membranes were incubated at room temperature for 1 h with secondary antibodies (1:5000, SA00001-1/SA00001-2, Proteintech, Chicago, IL, USA) diluted in 5% skim milk. Following three 5 min washes in TBST, the proteins were visualized by ECL (WBKLS0500, Millipore).

For immunoprecipitation, cells were lysed in cell lysis buffer (20 mM Tris-HCl pH 7.5, 150 mM NaCl, 1% Triton, 1 mM EGTA, 1 mM Na_2_EDTA, 2.5 mM sodium pyrophosphate, 1 mM β-glycerophosphate, 1 mM Na_3_VO_4_ and 1 mg/ml leupeptin) for 1 h at 4°C and then centrifuged at 12,000 rpm for 20 min. The supernatant was precleared with protein A/G, followed by incubation with 2 ml of primary antibody overnight at 4°C. Thirty microliters of protein A/G bead slurry (GE Healthcare) was added for an additional hour prior to an extensive wash in cell lysis buffer. After being washed, the pellets were boiled in SDS sample buffer for 5 min, and the immunoprecipitates were analyzed by Western blot as described above.

### Plasmid construction and site-directed mutagenesis

Human *DAX-1* complementary DNA (cDNA) was amplified from human testicle cDNA (636533, Takara, Japan) by PCR with the following primers: 5’- GGAATTCGCCACCATGGCGGGCGAGAACCACCA -3’ (forward) and 5’- CTTGTGGATCCCACATGACTTTATATCTTTGTACAG -3’ (reverse). The PCR product was subcloned into the EcoR1/BamH1 sites of a pcDNA3.1-HA expression vector (Invitrogen, Carlsbad, CA, USA). Site-directed mutagenesis was performed to generate *DAX-1* expression plasmids bearing the R51K, C104W, A242V, E256Q, V385L, or I427V mutation, as described previously [22]. DNA sequencing was performed to confirm the introduced mutations. The PCR primers used for site-directed mutagenesis and plasmid construction are shown in S2 Table.

### Luciferase assay

Luciferase analysis was performed as described previously with some modifications [23]. HeLa cells (ATCC, Manassas, VA, USA) were cultured in Dulbecco’s Modified Eagle’s Medium (Gibco BRL, Gaithersburg, MD, USA) supplemented with 10% fetal bovine serum, 100 U/ml penicillin and 100 μg/ml streptomycin at 37°C, 95% humidity and 5% CO_2_. Cells were seeded in 24-well tissue culture plates for 24 h prior to transfection. Equivalent amounts (100 ng) of DAX-1 (wild type [WT] or mutant) expression plasmids were cotransfected with mouse mammary tumor virus long terminal repeat (pMMTV-LUC) plasmids (100 ng) and an AR expression vector (10 ng) into HeLa cells using Lipofectamine 2000 (Invitrogen, Carlsbad, CA, USA), according to the manufacturer's instructions. Cells were treated with or without 100 nM testosterone after 6 h of transfection and harvested at 24 h after treatment. Firefly and Renilla luciferase expression was assessed using a Dual Luciferase Reporter Assay System (E1910, Promega, Madison, WI). Renilla luciferase activity was normalized to that of firefly luciferase. After normalization for transfection efficiency, induction factors were calculated as the ratio of the average of the luciferase value for the testosterone-stimulated samples vs. the non-testosterone-stimulated (ethanol vehicle-treated) samples.

### Statistical analysis

All experiments were repeated at least three times. Data were expressed as the mean ± SD. SPSS 17.0 statistical software was used for statistical analysis. Student’s t-test was used to compare the difference in means between the two groups. A p < 0.05 was considered to be statistically significant.

## Results

### Identification of DAX-1 mutation in patients with secretory azoospermia

**Fig 1:**
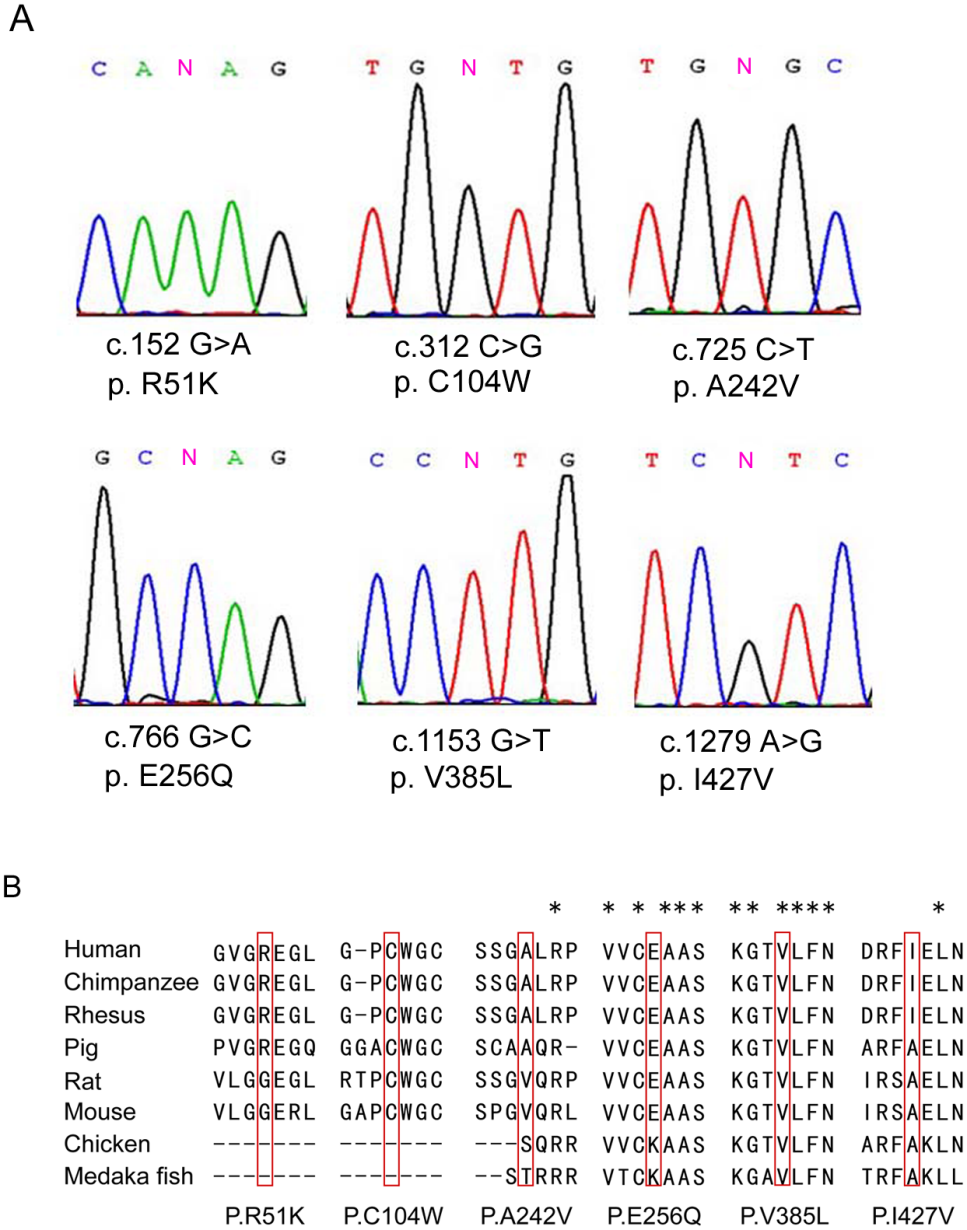
Six missense mutations in *DAX-1* identified in patients with secretory azoospermia. (A) Chromatogram traces from Sanger sequencing, showing the validated missense mutations. (B) Evolutionary conservation of amino acids affected by the missense mutations. Multiple protein alignments were performed with MegAlign (Demonstration System DNASTAR, Inc.). The identification numbers of the DAX-1 protein were as follows: human (NP_000466.2), chimpanzee (XP_520991.2), rhesus (XP_002806222.1), pig (NP_999552.1), rat (NP_445769.1), mouse (NP_031456.1), chicken (NP_989924.1), and Medaka fish (NP_001104259.1). The mutant alleles are boxed, and the star (*) indicates the conserved residue.

To examine whether *DAX-1* genetic defects are associated with secretory azoospermia, we screened for *DAX-1* exonic mutations in 776 secretory azoospermia patients and 709 men with proven fertility using massively parallel sequencing technology. As shown in Table 1, six missense mutations and six synonymous mutations were detected in *DAX-1*. All of the mutations except for c.498 G>A, c.376 G>A and c.114 C>T were not present in either dbSNP135 database or 1000 Genome Project dataset and were not identified in the 709 normal controls. The six novel missense mutations (c.152 G>A, c.321 C>G, c.725 C>T, c.766 G>C, c.1153 G>T and c.1279 A>G) were further confirmed by Sanger sequencing (Fig 1A). Alignment of the amino acid sequence of DAX-1 to its orthologs in different species showed that the V385L mutation affected a highly conserved amino acid (Fig 1B). Bioinformatic assessment of the variants indicated that the mutations C104W, E256Q and V385L were possibly damaging to the protein predicted by both Polyphen 2.0[24] and MutationTaster[25] (Table 2).

**Table 1:** *DAX-1* mutations and SNPs identified in the secretory azoospermia patients and controls.

No.	Position	Nucleotide change	Amino Acid Change	Patient (n = 776)	Fertile men (n = 709)	dbSNP135
Missense mutations						
1	30322830	c.1279 A>G	p.I427V	1	0	
2	30326328	c.1153 G>T	p.V385L	1	0	
3	30326715	c.766 G>C	p.E256Q	1	0	
4	30326756	c.725 C>T	p.A242V	2	0	
5	30327169	c.312 C>G	p.C104W	1	0	
6	30327329	c.152 G>A	p.R51K	3	0	
Synonymous mutation						
7	30326903	c.578 C>G	None	0	1	
8	30326983	c.498 G>A	None	550	539	rs2269345
9	30327105	c.376 G>A	None	26	26	rs193205940
10	30327319	c.162 G>A	None	1	0	
11	30327367	c.114 C>T	None	99	83	rs6150
12	30327383	c.98 G>T	None	1	0	

**Table 2:** List of missense mutations predicted by PolyPhen 2.0 and Mutation Taster.

No.	Nucleotide Change	Amino Acid Change	Polyphen	MutationTaster
1	c.152 G>A	p.R51K	Possibly damaging	polymorphism
2	c.312 C>G	p.C104W	Probably damaging	disease causing
3	c.725 C>T	p.A242V	Benign	polymorphism
4	c.766 G>C	p.E256Q	Probably damaging	disease causing
5	c.1153 G>T	p.V385L	Possibly damaging	disease causing
6	c.1279 A>G	p.I427V	Benign	polymorphism

### Interaction between DAX-1 mutants and AR

**Fig 2:**
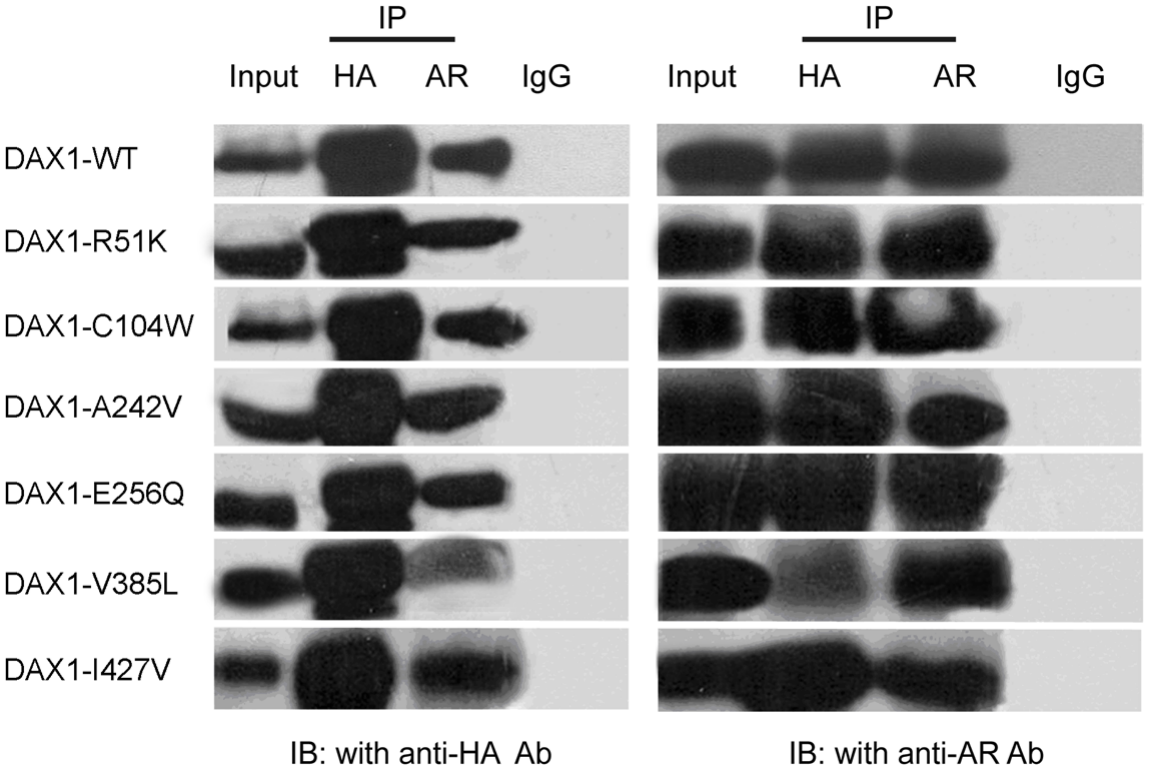
Interactions between DAX-1 mutants and AR. HeLa cells were transfected with expression vectors for AR, DAX-1 WT, DAX-1 R51K, DAX-1 C104W, DAX-1 A242V, DAX-1 E256Q, DAX-1 V385L, and DAX-1 I427V as indicated. DAX-1 and AR were immunoprecipitated (IP) with an anti-HA antibody and AR antibody, respectively. Then, the immunocomplexes were analyzed by SDS-PAGE and Western blotting (WB) analysis using anti-HA and anti-AR antibodies as indicated.

DAX-1 inhibits the transcriptional activity of AR through protein-protein interactions [18–20]. To determine whether the identified missense mutations in DAX-1 affect its ability to bind to AR, WT and mutated DAX-1 constructs were transfected into HeLa cells with an AR plasmid. The results showed that all DAX-1 R51K, C104W, A242V, E256Q, and I427V mutants co-immunoprecipitated with AR similar to DAX-1 WT but that V385L was weakly bound to AR (Fig 2). Collectively, these results indicated that the V385L mutation affected the interaction between DAX-1 and AR.

### Effects of DAX-1 mutations on AR function

**Fig 3:**
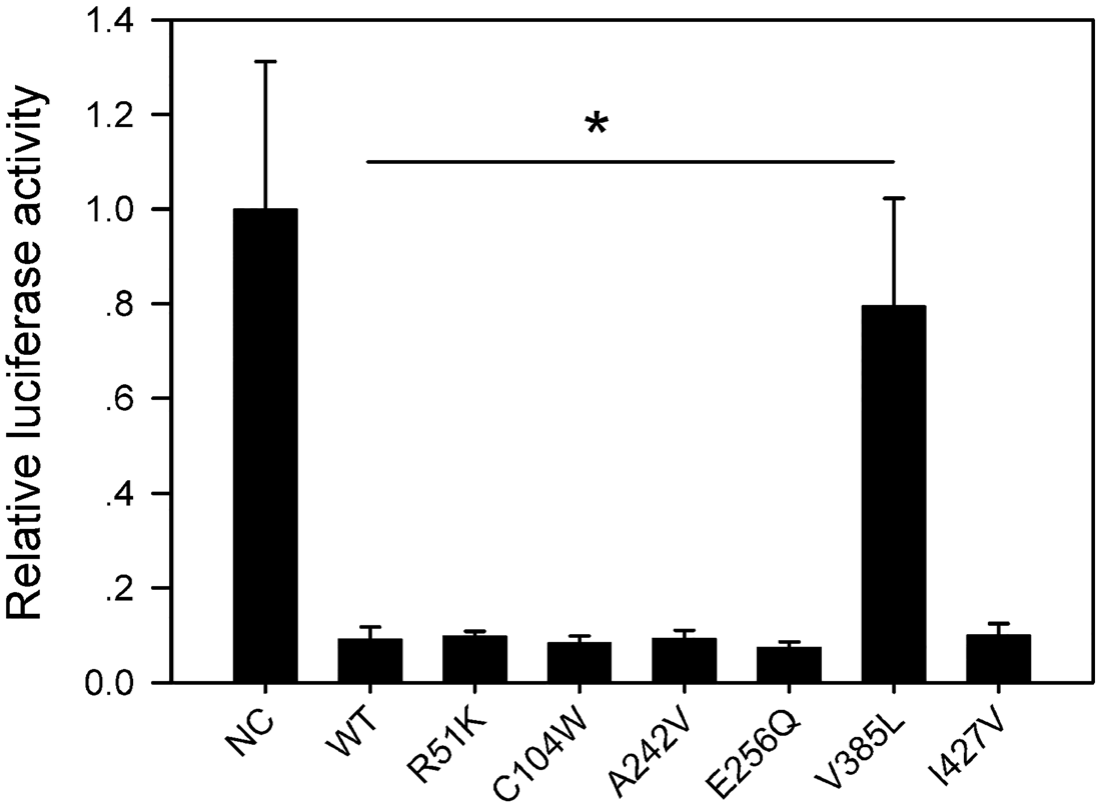
The effect of the DAX-1 V385L variant on AR function. A WT or mutant DAX-1 expression vector was cotransfected with an AR expression vector and a testosterone-inducible pMMTV-LUC plasmid into HeLa cells, and luciferase activity was measured with or without testosterone treatment. Compared with the WT and the other mutants, the DAX-1 V385L mutant failed to repress the transcriptional activity of AR in the presence of testosterone. The results are presented as the fold-change of testosterone treated relative to that in the vehicle-treated control. NC: pcDNA3.1-HA were cotransfected with an AR expression vector and a testosterone-inducible pMMTV-LUC plasmid into HeLa cells. (* *p* < 0.05).

To test whether the six identified missense mutations in DAX-1 eliminate its repression of AR, HeLa cells were transfected with *DAX-1* expression vectors containing these mutations along with AR plasmids. We used an androgen-responsive luciferase reporter construct, namely pMMTV. In the absence of DAX-1, AR activated this reporter in an agonist-dependent fashion. The inhibition of AR by DAX-1 V385L was significantly reduced, while no changes were observed for the other mutants compared with the WT (Fig 3). These results indicated that V385L eliminated the repression of AR by DAX-1.

### Clinical data

**Fig 4:**
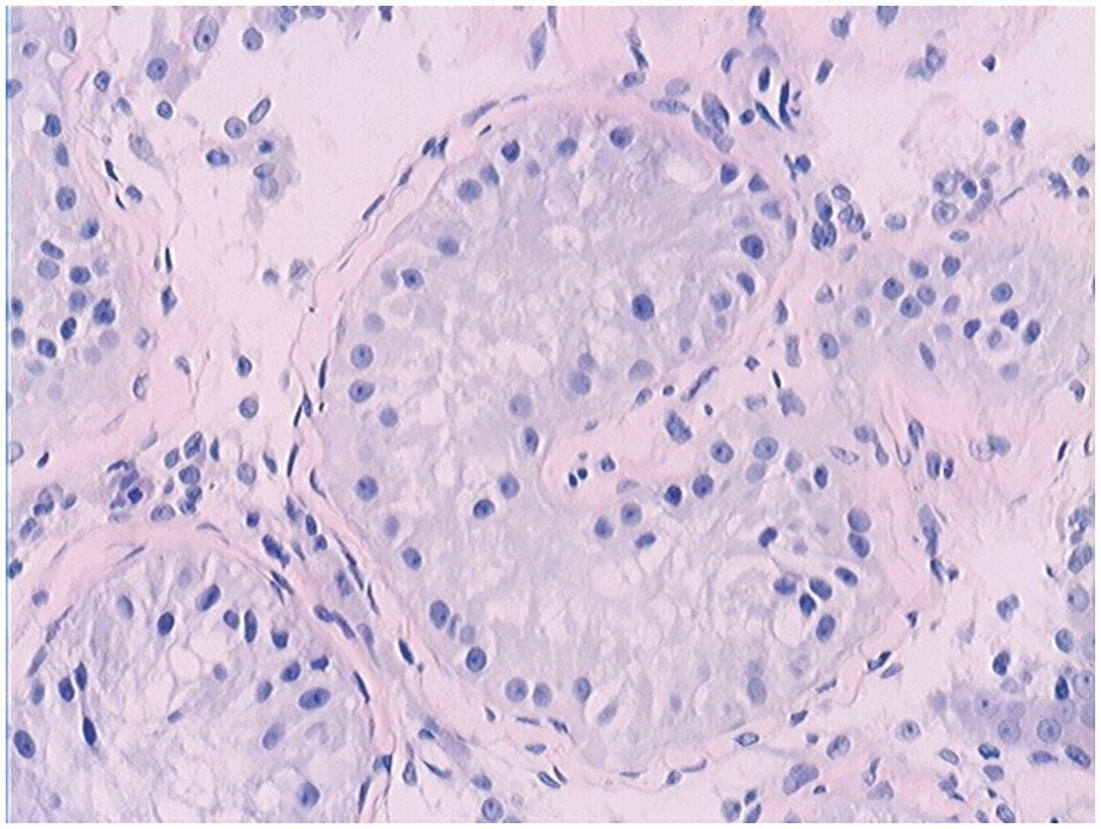
Testicular histology analysis of the patient with the V385L mutation by hematoxylin and eosin staining (Magnification: 400x). The spermatogenesis process was mainly blocked at the spermatocyte stage.

The clinical information of the patients with DAX-1 missense mutations is shown in Table 3. The patient with the V385L mutation had normal hormone levels and no relevant reproductive family history. The histology of the testes of this patient confirmed the diagnosis of non-obstructive azoospermia and showed an arrest of spermatogenesis at the spermatocyte stage (Fig 4).

**Table 3:** Clinical information of the patients with missense mutation in *DAX*-*1*

Nucleotide Change	Patient No.	Age	Testicular volume (ml)	FSH(mIU/ml) [1.5–12.5mIU/ml]	LH(mIU/ml) [1.7–8.6mIU/ml]	T(ng/ml) [2.5–8.0ng/ml]
c.152 G>A	24	37	6	NA	NA	NA
c.152 G>A	566	30	3	2.94	3.41	3.07
c.152 G>A	698	32	6	2.58	1.80	1.02
c.312 C>G	505	44	NA	2.82	3.95	1.32
c.725 C>T	33	34	2	NA	NA	NA
c.725 C>T	251	33	2	4.82	5.04	3.26
c.766 G>C	570	32	5	25.38	12.13	2.64
c.1153 G>T	628	31	15	3.24	4.28	2.84
c.1279 A>G	520	38	5	10.57	4.22	2.57

NA: Not available.

## Discussion

It is known that humans carrying mutations in the *DAX-1* locus often exhibit primary adrenal failure, hypogonadotropic hypogonadism and azoospermia. *Dax-1* knockout mice exhibit dysgenesis and degeneration of the testicular germinal epithelium until the complete loss of germ cells after 14 weeks, while the serum hormone levels, including the LH and FSH levels, in DAX-1 knockout mice are indistinguishable from those of wild-type mice, suggesting primary testicular failure rather than dysfunction at the pituitary level [8]. Therefore, it is possible that somatic mutations in DAX-1 might be present in a subset of patients with secretory azoospermia, normal hormone levels, and no relevant reproductive family history. In a previous study, no DAX-1 mutations were detected in 15 testicular biopsy samples from men with secretory azoospermia because of the limited sample size [26]. To date, no causative mutation has been identified in *DAX-1* for secretory azoospermia.

Previous Studies have shown that DAX-1 directly interacts with AR and represses its activity. AR is present in the cytoplasm in the absence of its ligand, but it is transported to the nucleus upon ligand binding to carry out its functions. Holter et al. have shown that cytoplasmic DAX-1 tethers AR in the cytoplasm in the presence of its ligand, preventing its translocation to the nucleus. Because both AR and DAX-1 are located on the X chromosome, all mutations affecting their functions yield a phenotype [18].

In this study, six novel missense mutations were detected in *DAX-1* in the patients with secretory azoospermia. Of these mutations, V385L affected the inhibition of transcriptional AR activation by DAX-1. *In vitro* transient transfection assays showed that this mutation affected the ability of DAX-1 to repress transcription of AR compared with wild-type DAX-1. These results indicate that aberrations of *DAX-1* might be intolerable and that the V385L mutation of *DAX-1* might be involved in impairment of human spermatogenesis. Unfortunately, the family of this patient was not available for genotype–phenotype correlations. Other variants of DAX-1 may also be pathogenic, causing malfunctions by restricting the inductive activities of testis-promoting factors, such as SRY and SF-1, thereby modulating their effects during testicular development [27, 28]. Thus, the additional variants of DAX-1 assessed in this study may also be pathogenic and cause malfunction due to the following reasons: (1) there are functional limitations of the pMMTV-LUC promoter, which is only responsive to more severe mutations but is not informative for mild mutations [29]; and (2) DAX-1 also represses the inductive activities of testis-promoting factors, such as SRY and SF-1, thereby modulating their effects during testicular development [27, 28].

In summary, a novel mutation (p. V385L) in DAX-1 was detected in a patient with secretory azoospermia with no personal or family history of X-linked adrenal hypoplasia congenita or adrenal insufficiency. This mutation potentially contributed to the onset of secretory azoospermia in this patient.

## Supporting Information

S1 Table Primers used for PCR and Sanger sequencing validation of *DAX-1* gene. (DOC)

S2 Table Primers used for site-directed mutagenesis construction. (DOC)
