# Supplementary material for: A Novel Mutation of DAX-1 Associated with Secretory Azoospermia
Source: PLoS One. 2015 Jul 24;10(7):e0133997. doi: 10.1371/journal.pone.0133997 (PMC4514677; doi:10.1371/journal.pone.0133997)
Supplement: S1 Table — (DOC) [file pone.0133997.s001.doc]

| **S1 Table. Primers using for PCR and sanger sequencing validation of *DAX-1* gene** | |
| --- | --- |
| Primer ID | Sequences (5'-3') |
| primer 1F | CCATGGCGGGCGAGAAC |
| primer 1R | GCCCGCCCGCCTGGTAG |
| primer 2F | CGGGCCACGGCGCTTCTGT |
| primer 2R | TCCCGCCGCCTGGTGGTGAG |
| primer 3F | CACCAGGCGGCGGGAGACC |
| primer 3R | GATAGCAGCCCACCCAGCAGAAGT |
| primer 4F | TTGGGTCTTGTTTAATTGGGATGA |
| primer 4R | TTTTGCCCACAGCTCTTTATTCTT |
