# Supplementary material for: A Novel Mutation of DAX-1 Associated with Secretory Azoospermia
Source: PLoS One. 2015 Jul 24;10(7):e0133997. doi: 10.1371/journal.pone.0133997 (PMC4514677; doi:10.1371/journal.pone.0133997)
Supplement: S2 Table — (DOC) [file pone.0133997.s002.doc]

| **S2 Table. Primers used for site-directed mutagenesis construction** | |
| --- | --- |
| Primer ID | Sequences (5'-3') |
| Mut1F- R51K | GAGCCCGGGGTGGGCAAAGAGGGGCTGCTGGGC |
| Mut1R- R51K | GCCCAGCAGCCCCTCTTTGCCCACCCCGGGCTC |
| Mut2F- C104W | CGACGCTGGGTCCGTGGTGGGGCTGTTCGTGCG |
| Mut2R- C104W | CGCACGAACAGCCCCACCACGGACCCAGCGTCG |
| Mut3F- A242V | GACACCTCCTCTGGTGTGCTGCGGCCGGTGGCG |
| Mut3R- A242V | CGCCACCGGCCGCAGCACACCAGAGGAGGTGTC |
| Mut4F- E256Q | TCCACAGGTGGTCTGCCAGGCAGCCTCAGCGGG |
| Mut4R- E256Q | CCCGCTGAGGCTGCCTGGCAGACCACCTGTGGA |
| Mut5F-V385L | CTACCTCAAGGGGACCTTGCTCTTTAACCCGGG |
| Mut5R-V385L | CCCGGGTTAAAGAGCAAGGTCCCCTTGAGGTAG |
| Mut6F-I427V | GCCCCATGACAGATTCGTCGAACTTAATAGTAC |
| Mut6R-I427V | GTACTATTAAGTTCGACGAATCTGTCATGGGGC |
